# Psychometric evaluation and adaptability of the simplified Chinese version of HOOS-12 in patients undergoing total hip arthroplasty

**DOI:** 10.1186/s12955-026-02491-2

**Published:** 2026-02-12

**Authors:** Mengyuan He, Lin Wu, Wei Gu, Dongfa Liao, Benjing Song, Hongbao Hu, Lin Cui, YidaAmu, Shihong Li, Yingchao Tang, Jianxiang Long, Li Yin, Qingyun Xie, Wei Wang

**Affiliations:** 1 Department of Orthopedics, The General Hospital of Western Theater Command PLA, Chengdu, 610083 China; 2 College of Medicine, Southwest Jiaotong University, Chengdu, 610031 China; 3 Department of Hepatobiliary, Chongqing Emergency Medical Center, Chongqing University Central Hospital, School of Medicine, Chongqing University, Chongqing, 400030 China; 4 Department of Orthopedics, The First Veterans Hospital of Sichuan Province, Chengdu, 610501 China

## Abstract

**Introduction:**

Considering the response burden of the full version of the HOOS scale, researchers developed a more concise HOOS-12 scale. Although the HOOS-12 has been verified to have good reliability, validity and responsiveness in most Western-country studies, it still needs further evaluation in other non-English populations. Therefore, our aim is to assess the content validity, internal consistency, structural validity, test-retest reliability, responsiveness, floor effect and ceiling effect of the Chinese version of HOOS-12 in the patient population whose native language is Chinese. The goal is to provide a high-quality evaluation tool for the global Chinese-using hip joint patient population, promote cross-cultural and cross-regional hip joint research, and ensure the comparability of the data.

**Method:**

Patients with chronic hip diseases scheduled for total hip arthroplasty were included in the study. Content validity was evaluated using expert review to assess item relevance. CFA was employed to validate the construct validity of the CHOO-12. Convergent and discriminant validity of the CHOOS-12 were assessed using the SF-36, OHS, and WOMAC scales as reference instruments. The first HOOS-12 scores were used to evaluate content validity, internal consistency, and structural validity. The second set of scores was utilized to assess test-retest reliability and ceiling/floor effects. The third set of scores was applied to evaluate responsiveness.

**Result:**

The CHOOS-12 demonstrated good content validity. No ceiling or floor effects were observed for the CHOOS-12 total scale or its pain, function, and quality of life subscales. The internal consistency among items was high for both the overall CHOOS-12 and all its subscales. CFA indicated satisfactory performance for the pain dimension, though the unidimensionality of the function and quality of life dimensions was challenged. The CHOOS-12 scale exhibited good convergent and discriminant validity. With overall ES and SRM values of 2.08 and 2.42 respectively, the CHOOS-12 also demonstrated good responsiveness.

**Conclusion:**

CHOOS-12 demonstrated good reliability and validity in this study. In terms of construct validity, the pain subscale performed well, although the unidimensionality of the PF and QOL subscales was challenged, the core items still had measurement potential. Future research needs to conduct further validation in larger and more diverse samples.

## Introduction

Many diseases can lead to the loss of hip joint function, including osteonecrosis of the femoral head (ONFH), hip osteoarthritis (hip OA), Perthes disease, and rheumatoid arthritis (RA). The loss of hip joint function is mainly manifested as limited internal rotation, flexion, and external rotation of the hip joint, accompanied by severe pain during weight-bearing activities. These hip joint diseases develop slowly, have a long course, and greatly affect patients’ work and daily life [1]. Hip joint diseases are extremely common worldwide. In China, the number of patients with ONFH and hip OA alone is quite large. According to a survey, the number of new ONFH cases in China each year is between 100,000 and 200,000 [2]. According to a study published in 2025, the global population is facing a public health threat of an increasing number of patients with hip OA. In the next 50 years, the number of deaths and disability-adjusted life years cases will still increase [3]. Chronic hip joint diseases impose a great burden on the public health system. In this context, developing and applying efficient and reliable patient-reported outcome tools is crucial for precisely assessing patients’ feelings, guiding clinical decisions, and evaluating treatment effects.

Patient-reported outcome measures (PROMs) were originally developed as important measurement tools to quantify subjective experiences, standardize evaluations, and enhance the reliability and validity of assessments [4]. These instruments directly reflect patients’ perceptions of their own health status, functional ability, and treatment responses, without interpretation by others, thus providing an objective representation of their personal perspectives. Since the 1980s, clinicians and researchers have conducted a series of studies on patient-reported health-related outcomes (HR-PROs) [5]. PROMs are now widely used in orthopedics, playing significant roles in assessing psychological status [6], monitoring treatment outcomes [7], and improving postoperative pain management [8] among orthopedic patients. Several specific instruments are available for evaluating hip joint function, such as The Western Ontario and McMaster Universities Arthritis Index (WOMAC) [9], Oxford Hip Score(OHS) [10], and Hip disability and Osteoarthritis Outcome Score (HOOS) [11]. Among these, the HOOS was adapted from the WOMAC by Emilie et al. [12]. It is designed to assess the impact of hip osteoarthritis on daily life, evaluate functional status before and after total hip arthroplasty (THA), and monitor patients with other hip conditions such as hip dysplasia and osteonecrosis of the femoral head. The HOOS consists of 40 items across five subscales: Pain, Other Symptoms, Function in Daily Living, Function in Sport and Recreation, and Hip-Related Quality of Life. It is recognized for its comprehensiveness, flexibility, and strong reliability, validity, and responsiveness, and has been translated into multiple languages for global use [13–15]. However, the HOOS has certain limitations. Its length may lead to respondent fatigue and reduced patience during completion, potentially affecting measurement accuracy [16]. Moreover, the HOOS yields only a composite score, which limits its ability to capture specific changes in pain, function, and quality of life. To address these issues, Gande et al. refined the HOOS by selecting and reducing items from the pain, function, and quality of life subscales, ultimately developing the 12-item Hip disability and Osteoarthritis Outcome Score (HOOS-12) [17]. Compared to the original HOOS, the HOOS-12 maintains a focused assessment of hip pain, function, and quality of life while providing sufficient coverage across these domains to form a comprehensive measure of overall hip impact. It retains high reliability, validity, and responsiveness [18], with the added advantage of being suitable for rapid clinical assessment, making it more effective in reflecting the impact of hip dysfunction in patients with chronic hip conditions. The HOOS-12 was originally developed in English and has since been linguistically and culturally adapted and validated in several countries, including France [19], Germany [20], and Australia [21], where it demonstrated strong psychometric properties. However, at present, there is a lack of research on the application of HOOS-12 in the Chinese-speaking population. With a population of over 1.4 billion, China has a substantial number of individuals affected by chronic hip conditions, highlighting the need for a tool like the HOOS-12 in the assessment of Chinese patients with hip disorders. However, to ensure scientific rigor and comparability of results, the instrument must undergo systematic cross-cultural adaptation and rigorous validation of its reliability and validity before it can be reliably applied in the Chinese population.

Therefore, the aim of this study is to translate the original HOOS-12 into the Chinese version of HOOS-12 scale, and conduct tests among the Chinese-speaking patients with hip joint diseases to evaluate its reliability, validity and responsiveness. To provide a high-quality evaluation tool for the global Chinese-speaking hip joint patient population and promote cross-cultural and cross-regional hip joint research worldwide.

## Methods

According to the diagnostic research evidence grading standard of the Oxford Centre for Evidence-Based Medicine, this study is designed as a single-center observational validation study, and the evidence grade is: III.

### Translation and cross-cultural adaptation

The translation of the scale in this study followed established cross-cultural adaptation guidelines [22, 23]. It is worth noting that in cross-cultural translation, since the question stems of each question in the original version were very concise, in order to increase the response rate and avoid survey errors caused by misunderstandings, we made the descriptions of the question stems more detailed. Additionally, considering that some of the action scenarios described in the questions were relatively complex, we were concerned that some patients might have difficulty understanding (especially elderly patients), so we reminded all participants at the beginning of the questionnaire that they could consult the medical staff present. Moreover, during each questionnaire survey, there were medical staff present to provide consultation services.

### Patients and data acquisition

The target population consists of patients with chronic hip joint diseases who were treated at the General Hospital of the Western Theater Command from March 2023 to March 2024, and whose native language is Chinese and underwent THA. Inclusion criteria: (1) Age > 18 years, with the right to give informed consent; (2) Able to independently read and complete the questionnaire in Chinese; (3) Diagnosed with hip joint diseases based on clinical manifestations and imaging examinations. Exclusion criteria: (1) Previous history of femoral neck fractures or hip joint surgeries; (2) Acute musculoskeletal injury history in the hip joint area within the past 3 months; (3) Other chronic inflammatory diseases in the hip joint area that may affect hip joint function. When developing and validating the original HOOS-12, the questionnaire completion method was for patients to complete the questionnaire in the doctor’s office or at home before, 6 months after, and 12 months after THA surgery, in the form of a paper questionnaire or online filling [17, 18]. The validation of the original HOOS-12 involved filling out the scale three times before, 6 months after, and 12 months after THA surgery. We required patients to independently complete the CHOOS-12 (HOOS-JR, HOOS-PS were also completed simultaneously for other studies, used for other research), OHS, 36-Item Short Form Health Survey (SF-36) [24], and WOMAC 6 scales on the first day of admission. One week after the first questionnaire completion, the second scale test was conducted. Finally, considering the data collection method of the original HOOS-12 and the internet penetration rate of the elderly population in China, a third scale test was conducted 6 months later, using outpatient follow-up or telephone interviews. This study adheres to the Helsinki Declaration, and all volunteers were required to carefully read and sign the informed consent form. At the same time, the hospital ethics committee approved this clinical study, and the ethical approval number is: 2024EC1-ky020.

### Instruments

**Fig. 1:**
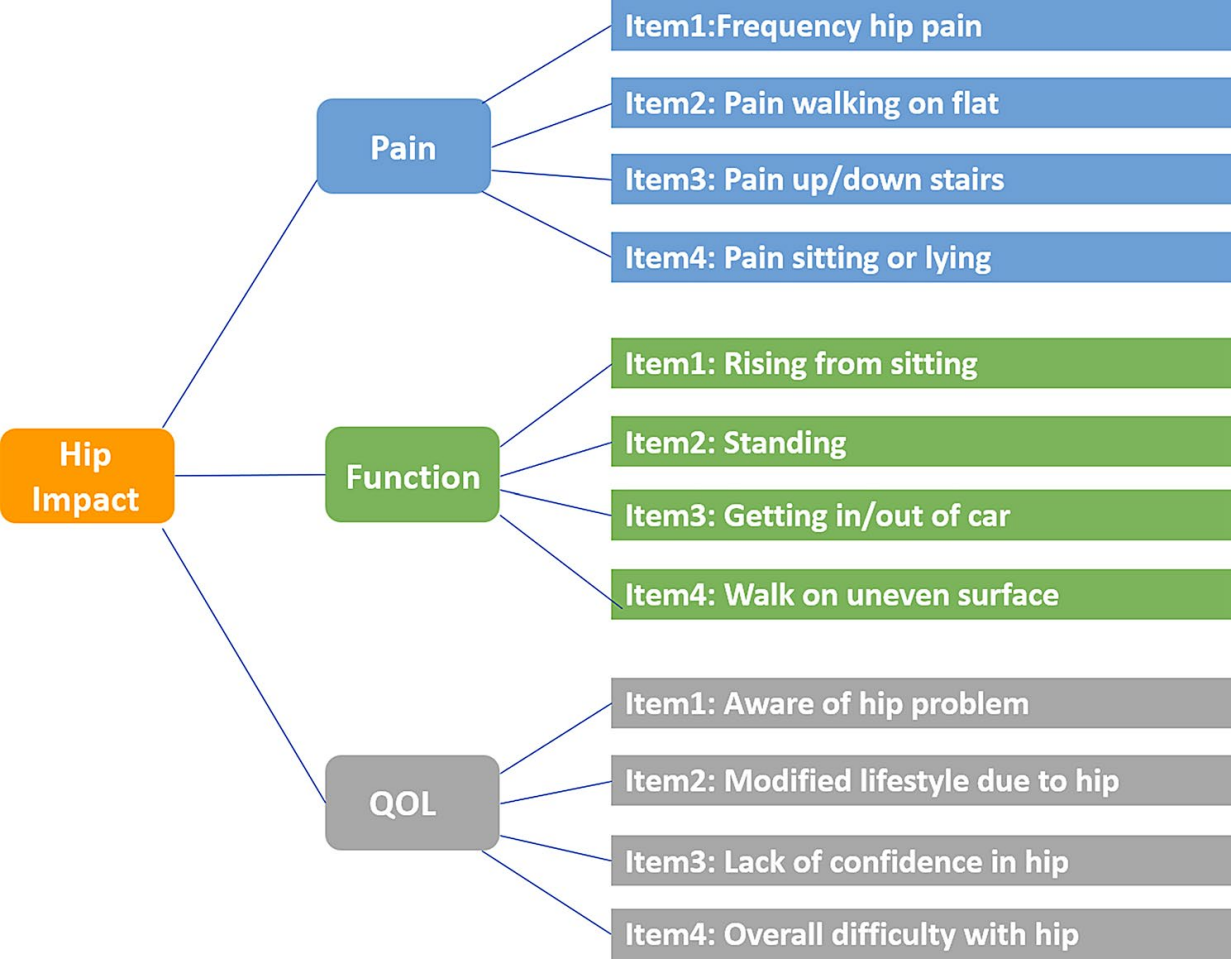
HOOS-12 measurement model

The original HOOS-12 scale comprises three subscales with a total of 12 items: four in the Pain subscale, four in the Physical Function (PF) subscale, and four in the Quality of Life (QOL) subscale(Figure1). In the validation of the original HOOS-12, the researchers used the cumulative scoring method, that is, in a scale, the scores of each item are directly added to obtain a preliminary total score. Subsequently, the scores are converted to a percentage system, where 0 represents the worst and 100 represents the best measured score [18]. Since HOOS-12 was developed based on HOOS and contains multiple potential variables, the researchers directly conducted confirmatory factor analysis (CFA) on each of the three subscales of HOOS-12 [25]. The results showed excellent construct validity, with all subscales demonstrating a comparative fit index (CFI) > 0.8, Tucker–Lewis index (TLI) > 0.8, root mean square error of approximation (RMSEA) < 0.08, and factor loadings all > 0.7. In the subsequent validation, the researchers determined the convergent validity and discriminant validity of the HOOS-12 scale by comparing it with the full version of the HOOS scale and the SF-36 scale [18]. The response rate of the original HOOS-12 was 96.5–97.6%, and the internal consistency was > 0.7. When compared with HOOS and HOOS-JR, the floor effect of the total HOOS-12 scale and its sub-scales was less than 2% before THA, 0.2% before THA surgery, and a significant ceiling effect occurred in almost all indicators after THR surgery. Regarding pain, the ceiling effect of the HOOS-12 pain scale was 49% to 50% at 6 and 12 months after surgery, the PF scale was 33%-41%, and the QOL scale was 17.4–20.1%. The ceiling effect of the overall HOOS-12 score in the post-THA ceiling effect was the lowest, at 12%-14%. In contrast, the ceiling effects of other scales (such as HOOS and JR) were 27%-36%, indicating that the overall score of HOOS-12 was more advantageous than other comprehensive assessment indicators in differentiating patients with different hip joint health levels. At 6 and 12 months after total hip arthroplasty, the effect size (ES) of the HOOS-12 pain scale was slightly higher than that of the pain scale of HOOS, while the standardized response mean (SRM) was similar. The ES and SRM of the PF scale of HOOS and the QOL scale of HOOS were similar. The ES of the motion/recreation scale of HOOS was higher but the SRM was lower than other functional scales. The ES of HOOS-PS was the lowest among all scales. The ES of the quality of life scale of HOOS-12 was 2.77 and 3.04 at 6 and 12 months, respectively. The ES of the total score of HOOS-12 (2.90–3.16) was slightly higher than that of HOOS and JR (2.34–2.56), and the SRM of the total score of HOOS-12 (2.31–2.64) was also higher than that of HOOS and JR (2.01–2.21). This indicates that the HOOS-12 scale, especially its pain scale and total score, performed well in evaluating the effect of total hip arthroplasty, and its sensitivity was superior to or equivalent to other scales.

### Statistical analysis

The consensus-based health measurement tool selection criteria (COSMIN) methodology was used to evaluate the measurement characteristics of the scale [26]. The specific Quality criterias are shown in Table 1.

**Table 1:** The measurement properties and evaluation criteria of the CHOOS-12 scale

Property	Definition	Quality criteria
Content validity	The extent to which the domain of interest is comprehensively sampled by the items in the questionnaire	I-CVI S-CVI/UA
Internal consistency	The extent to which items in a (sub)scale are intercorrelated, thus measuring the same construct	Cronbach’s alpha(s)
Construct validity	The extent to which scores on a particular questionnaire relate to other measures in a manner that is consistent with theoretically derived hypotheses concerning the concepts that are being measured	CFA Spearman’s correlation coefficient (r_s_) Hypothesis testing
Reproducibility	The reproducibility of a scale refers to the degree of consistency or stability of the results when the same scale is used to measure the same group of subjects at different time points (or by different assessors) under the same measurement conditions.	ICC
Responsiveness	The ability of a questionnaire to detect clinically important changes over time	ES SRM
Floor and ceiling effects	The number of respondents who achieved the lowest or highest possible score	/

I-CVI, content validity index of the item level; S-CVI, Scale-level Content Validity Index; CFA, Confirmatory Factor Analysis; ICC, intraclass correlation coefficient; ES, effect size; SRM, standardized response means

### Content validity

In this study, content validity indices were used to quantify the relevance of scale items to the measured content dimensions. Content validity indices are divided into two categories: the Item-level Content Validity Index (I-CVI), which evaluates the content validity of individual items, and the Scale-level Content Validity Index (S-CVI), which assesses the content validity of the entire scale [27]. In the expert evaluation questionnaire for content validity, experts were asked to judge the relevance of each item to its corresponding content dimension. Ratings were then dichotomized: scores of 1 or 2 indicated that the item was not relevant to the content dimension and poorly representative of the measured concept; scores of 3 or 4 indicated that the item was relevant and representative. The I-CVI for each item was calculated as the number of experts rating it 3 or 4 divided by the total number of participating experts. Lynn proposed criteria for interpreting I-CVI: when the number of experts is five, the I-CVI should be 1.00, meaning all experts agree that the item is well-related to the measured concept for the item to be considered having good content validity [28]. The S-CVI/UA (universal agreement) is the proportion of items rated 3 or 4 by all experts. An S-CVI/UA not less than 0.8 indicates good content validity of the scale [27]. Our panel for evaluating the relevance of the translated scale consisted of two associate chief orthopedic surgeons, one associate chief rehabilitation physician, one associate chief statistical technician, and one linguistics specialist.

### Internal consistency

Internal consistency is an indicator used to measure the degree of homogeneity among the items in a scale, ensuring that these items measure the same concept. Internal consistency is typically evaluated using Cronbach’s α value. Generally, it is considered that when α is greater than 0.7, the scale has acceptable internal consistency [29].

### Construct validity

The original HOOS-12 has excellent construct validity. In a cross-cultural context, the measurement model of a known structure can also be tested for its applicability using CFA [30]. To obtain reliable data, a sufficient sample size is required for data analysis. According to the rule of thumb, for a scale, the sample size should be 10 times the number of items when conducting CFA. HOOS-12 has 12 items, so the sample size should be at least > 120 [29, 31]. However, it is worth noting that although the sample size meets the minimum requirement for CFA, for a short subscale consisting of only 4 items, the sample size is relatively limited, which may limit the robustness of model fit. At the same time, this study selected OHS, WOMAC, and SF-36 as the control scales for the convergent validity and discriminant validity evaluation of CHOOS-12.OHS is a specific scale specifically designed for hip joint diseases, while WOMAC is a scale targeting lower limb osteoarthritis (including the hip and knee joints), and SF-36 is a general scale with wide applicability [32–34]. We hypothesize that the results of the CHOOS-12 questionnaire have a good correlation with the physiological function scales in OHS and WOMAC (physiological function, pain, and joint stiffness) and the physiological function subscale in SF-36 (physiological function, physiological role, physical pain, and overall health). The correlation with the psychological subscale of SF-36 (energy, social function, emotional role, and mental health) is relatively poor. Moreover, the correlation between CHOOS-12 and OHS should be stronger than that between WOMAC and the subscales of SF-36. We analyzed the Spearman correlation coefficients (rs) between CHOOS-12 and OHS, WOMAC, and SF-36, and tested whether they fell within the preset interval to verify the hypothesis. When at least 75% of the preset hypotheses are satisfied, it proves that CHOOS-12 has good convergent validity and discriminant validity [29].

### Reproducibility

Reproducibility focuses on the similarity of results obtained from repeated measurements in individuals. It is important to distinguish between reliability and agreement. Agreement describes the range of variation in repeated measurement scores and is calculated using the standard error of measurement (SEM): SEM = SD × √(1 - ICC). The standard deviation (SD) of all patients’ scale scores at the first assessment is used to calculate SEM. The minimal detectable change (MDC) reflects the smallest individual change that can be interpreted as a true change. At the individual level, MDC = SEM × 1.96 × √2; at the group level, MDC = SEM × 1.96 × √2 / √n. Systematic errors between the first two measurements were determined using Bland-Altman plots. For evaluative purposes (e.g., distinguishing clinically important changes from measurement error), a smaller measurement error is required. Reliability, on the other hand, focuses on the degree to which patients can be distinguished in the presence of measurement error. Test-retest reliability was evaluated using the intraclass correlation coefficient (ICC), with ICC > 0.8 indicating good reliability. The time interval between repeated measurements should be long enough to avoid recall bias but short enough to ensure no significant changes in the patient’s condition. To assess the test-retest reliability of the CHOOS-12, patients were asked to complete the questionnaire twice, one week apart, and the answers were compared [29].

### Responsiveness

In this study, the responsiveness of the CHOOS-12 was evaluated by comparing the scale results before treatment and six months after surgical treatment. The surgical treatment was THA, and the two indicators of responsiveness were the SRM and ES [35]. SRM was calculated by dividing the mean change between time points by the standard deviation of that change. ES was calculated as the mean change in treatment outcomes over six months divided by the standard deviation of the pre-treatment CHOOS-12 scores. SRM and ES values exceeding 0.80 were considered indicative of a large effect size.

### Floor/ceiling effect

The floor effect/ceiling effect can be used to assess the comprehensiveness of a scale. If more than 15% of the respondents obtain the lowest or highest score respectively, it can be considered that there is a floor effect/ceiling effect [36]. If such an effect exists, it indicates that the content validity is limited. Therefore, patients with the lowest or highest scores cannot be distinguished from each other, which reduces the reliability. Additionally, the response ability will also be restricted because changes cannot be measured in these patients. If the scale does not have a floor effect or ceiling effect, it is considered that the comprehensiveness of the scale is better [29].

Data were organized and reliability was tested using the Social Science Statistical Software Package 25.0 (SPSS, Chicago, IL, USA). Structural validity was evaluated using the Scientific Statistical Software Package 28.0 (Amos, Chicago, IL, USA). The mean was expressed in standard deviation (SD). The ICC value was represented by the 95% confidence interval (CI). A P value less than or equal to 0.05 was considered statistically significant.

## Result

### Patients

This study included a total of 142 patients with hip joint diseases. The average age was 58.09 ± 10.83 years, and the average BMI was 24.08 ± 3.36. The other demographic data are detailed in Table 2. All patients completed the first questionnaire survey. 138 patients (84 males and 54 females) completed the second questionnaire survey. Among the 4 patients who did not complete the second HOOS-PS questionnaire, 3 gave up surgical treatment for various reasons and 1 lost contact. Additionally, a total of 109 patients (73 males and 36 females) completed the third questionnaire survey. Therefore, the scale data of 142 patients were finally included in the internal consistency, measurement error and validity analysis of the CHOOS-12 scale, the scale data of 138 patients were used for the test-retest reliability analysis, and the scale data of 109 patients were used for the responsiveness analysis.

**Table 2:** Demographic and clinical characteristics of participants

Characteristics	Number (%) or Mean ± SD
Age (years)	58.09 ± 10.83
Range	23–85
< 50	20 (14.1%)
≥ 50	122(85.9%)
Diagnosis	
osteoarthritis	52(36.6%)
osteonecrosis of the femoral head	85(59.8%)
rheumatoid arthritis	2(1.4%)
Perthes’ disease	3(2.1%)
Gender	
female	55 (38.8%)
male	87 (61.2%)
Affected side	
right	77(54.2%)
left	65(45.7%)
BMI (Kg/m^2^)	24.08 ± 3.36
<18.5	9 (6.3%)
18.5~24.9	80 (56.3%)
25~29.9	45 (31.7%)
> 30	8 (5.6%)

BMI: body mass index

### Content validity

Five experts evaluated the correlation between the items of the CHOOS-12 scale and hip joint pain, functional impairment, and quality of life (Table 3). The results showed that all five experts agreed that the items of the CHOOS-12 scale had excellent correlation with the concepts to be measured by the scale. Therefore, the Kappa value of the full scale was calculated as 1, and S-CVI/UA = 1, indicating excellent content validity. After reviewing the questionnaire content, the experts all believed that the information obtained from these questions was sufficient to assess the impact of hip joint dysfunction and osteoarthritis. Therefore, it was not recommended to omit or add any questions. Additionally, 20 THA patients were selected for the pre-test of the predictive version of the CHOOS-12, and no feedback of misunderstanding or misinterpretation of the questionnaire was received. No missing questions were found in the CHOOS-12 scale recovered in the formal survey, and no ceiling effect or floor effect was observed (Table 4). Furthermore, no patients reported difficulties or misunderstandings in completing the CHOOS-12 questionnaire.

**Table 3:** Content validity of the C-HOOS-12 and subscales

Item	Experts Score					The number of experts with a score of 3 or 4	^a^I-CVI	^b^ *P*_C_	^c^K^*^	Evaluation
	Expert 1	Expert 2	Expert 3	Expert 4	Expert 5					
Pain1	4	4	4	4	4	5	1.00	0.00	1.00	Excellent
Pain2	4	4	4	4	4	5	1.00	0.00	1.00	Excellent
Pain3	4	4	4	4	4	5	1.00	0.00	1.00	Excellent
Pain4	4	4	4	4	4	5	1.00	0.00	1.00	Excellent
PF1	4	4	4	4	4	5	1.00	0.00	1.00	Excellent
PF2	4	4	4	4	4	5	1.00	0.00	1.00	Excellent
PF3	4	4	4	4	4	5	1.00	0.00	1.00	Excellent
PF4	4	4	4	4	4	5	1.00	0.00	1.00	Excellent
QOL1	4	4	4	4	4	5	1.00	0.00	1.00	Excellent
QOL2	4	4	4	4	4	5	1.00	0.00	1.00	Excellent
QOL3	4	4	4	4	4	5	1.00	0.00	1.00	Excellent
QOL4	4	4	4	4	4	5	1.00	0.00	1.00	Excellent

^a^ The content validity index of the item level, the proportion of experts rating an item as content-relevant

^b^ Random consistency probability

^c^ Cohen’s Kappa, a statistical measure of inter-rater agreement that accounts for chance

### Internal consistency

The total score of the first test of the CHOOS-12 questionnaire was 59.6 ± 17.3, and the score of the second test was 58.3 ± 17.7. The total scores of the three subscales for both tests can be seen in Table 4. The Cronbach’s α value of the CHOOS-12 scale was 0.876, indicating that the scale has excellent internal consistency (Table 4).

**Table 4:** Reliability related data and floor-ceiling effects of the C-HOOS-12 and subscales

	C-HOOS-12	Subscales		
		Pain	Physical Function	Quality of Life
Mean ± SD (T0) ^a^	58.3 ± 17.0	57.4 ± 20.0	58.5 ± 18.2	58.8 ± 19.4
Mean ± SD (T1) ^a^	58.3 ± 17.8	57.6 ± 22.2	58.2 ± 22.8	59.2 ± 23.3
Cronbach’s α	0.876	0.753	0.671	0.731
ICC (CI range)	0.897 (0.859–0.925)	0.783 (0.709–0.840)	0.784 (0.710–0.841)	0.788 (0.715–0.844)
MDC(I)^b^	15.13	25.86	23.48	24.79
MDC(G)^c^	1.27	2.17	1.97	2.08
SEM	5.46	9.32	8.46	8.93
Floor effect(T0) (%) ^d^	0	0	0	0
Ceiling effect(T0) (%) ^d^	0.7	2.1	2.1	1.4
Floor effect(T2) (%) ^d^	0	0	0	0
Ceiling effect(T2) (%) ^d^	0	12.8	8.3	13.7

^a^ The T0 was conducted at the beginning of this research, the T1 was conducted one week later, the sample size included in the test-retest reliability analysis was 138 cases and T2 was conducted six months later, the sample size included in the test-retest reliability analysis was 109 cases

^b^ The MDC value at an individual level;

^b^ The MDC value at the group level

^c^ Percentage of patients with the worst (floor effect) and the best (ceiling effect) score

^d^C-HOOS-12: Chinese version of Hip disability and Osteoarthritis Outcome Score − 12 short form; ICC: intraclass correlation coefficient; CI: confidence interval; SEM: standard error of measurement; MDC: minimal detectable change

### Construct validity

The CFA results (Table 5) show that the validity of the pain subscale of the pain scale is good, while the factor models of PF and QOL have poor fit. The standardized factor loadings of each item are shown in Table 5. The results of this study are basically consistent with the prior hypotheses (94.2%, 179/190) (Tables 6 and 7).

**Table 5:** The confirmatory factor analysis results of the sub-scales of CHOOS-12

	Pain	Physical Function	Quality of Life
^a^X^2^/df	0.298	4.647	9.161
^b^TLI	1.033	0.771	0.620
^c^CFI	1.00	0.924	0.873
^d^RMSEA	0.000	0.161	0.241
^e^CR	0.7593	0.693	0.7377
^f^SFL of item 1	0.657	0.583	0.720
SFL of item 2	0.804	0.738	0.779
SFL of item 3	0.504	0.670	0.597
SFL of item 4	0.676	0.391	0.429

^a^ χ²/df, Chi-square/Degrees of Freedom, values < 3 indicate good fit, < 5 acceptable;

^b^ Tucker-Lewis Index, values > 0.90 good, > 0.95 excellent;

^c^ Comparative Fit Index, values > 0.90 good, > 0.95 excellent;

^d^ Root Mean Square Error of Approximation, values < 0.08 acceptable, < 0.05 good;

^e^ Composite Reliability, values > 0.70 indicate satisfactory reliability

^f^ Standardized factor loadings for all items are presented, with values > 0.50 generally considered acceptable, > 0.70 very good

**Table 6:** Construct validity of the C-HOOS-12

Spearman’s correlation coefficient (*r*_s_) ^a^	C-HOOS-12	Subscales		
		Pain	Physical Function	Quality of Life
**OHS**	-0.793 (*P* < 0.001)	-0.745 (*P* < 0.001)	-0.740 (*P* < 0.001)	-0.553 (*P* < 0.001)
**WOMAC**				
Physical function	0.760 (*P* < 0.001)	0.679 (*P* < 0.001)	0.794 (*P* < 0.001)	0.509 (*P* < 0.001)
Pain	0.675 (*P* < 0.001)	0.662 (*P* < 0.001)	0.610 (*P* < 0.001)	0.456 (*P* < 0.001)
Stiffness	0.600 (*P* < 0.001)	0.634 (*P* < 0.001)	0.603 (*P* < 0.001)	0.372 (*P* < 0.001)
**SF-36**				
Physical Function	-0.639 (*P* < 0.001)	-0.602 (*P* < 0.001)	-0.569 (*P* < 0.001)	-0.524 (*P* < 0.001)
Role-Physical	-0.543 (*P* < 0.001)	-0.517 (*P* < 0.001)	-0.484 (*P* < 0.001)	-0.436 (*P* < 0.001)
Bodily Pain	-0.540 (*P* < 0.001)	-0.572 (*P* < 0.001)	-0.563 (*P* < 0.001)	-0.316 (*P* < 0.001)
General Health	-0.464 (*P* < 0.001)	-0.441 (*P* < 0.001)	-0.472 (*P* < 0.001)	-0.324 (*P* < 0.001)
Vitality	-0.245 (*P* = 0.003)	-0.264 (*P* = 0.001)	-0.325 (*P* < 0.001)	-0.123 (*P* = 0.144)
Social Function	-0.401 (*P* < 0.001)	-0.328 (*P* < 0.001)	-0.304 (*P* < 0.001)	-0.410 (*P* < 0.001)
Role-Emotional	-0.104 (*P* = 0.219)	-0.163 (*P* = 0.053)	-0.111 (*P* = 0.189)	-0.035 (*P* = 0.678)
Mental Health	-0.230 (*P* = 0.006)	-0.263 (*P* = 0.002)	-0.295 (*P* < 0.001)	-0.044 (*P* = 0.602)

^a^ Calculated by the Spearman’s correlation coefficient (*r*_*s*_) of the C-HOOS-12 with OHS, WOMAC and SF-36

C-HOOS-12: Chinese version of Hip disability and Osteoarthritis Outcome Score − 12 short form; WOMAC: Western Ontario and McMaster Universities Osteoarthritis Index; SF-36: Short-Form 36

**Table 7:** The specific content of hypothesis test for construct validity

Hypotheses setting ^a, b^	C-HOOS-12	Subscales		
		Pain	Physical Function	Quality of Life
**OHS**	*r*_*s*_ > other scales (11/11)	*r*_*s*_ > other scales (11/11)	*r*_*s*_ > other scales (10/11)	*r*_*s*_ > other scales (11/11)
**WOMAC**				
Physical function Pain Stiffness	*r*_*s*_ > SF-36 (23/24)	*r*_*s*_ > SF-36 *r*_*s*_ with Pain > other subscales (25/26)	*r*_*s*_ > SF-36 *r*_*s*_ with Physical function > other subscales (26/26)	*r*_*s*_ > SF-36 (19/24)
**SF-36**				
Physical Function	*r*_*s*_ with PCS > MCS (16/16)	*r*_*s*_ with Bodily Pain > other subscales (6/7)	*r*_*s*_ with Physical Function > other subscales (7/7)	*r*_*s*_ with PCS > MCS (14/16)
Role-Physical				
Bodily Pain				
General Health				
Vitality				
Social Function				
Role-Emotional				
Mental Health				

^a^ The *r*_*s*_ is Calculated by the Spearman’s correlation coefficient of the C-HOOS-12 with OHS, WOMAC and SF-36

^b^ The correlation coefficient between the scales was compared with the 190 hypotheses set before the study to calculate the ratio conforming to the hypotheses. The denominator in brackets is the total number of corresponding hypotheses, and the numerator is the total number conforming to the hypotheses

C-HOOS-12: Chinese version of Hip disability and Osteoarthritis Outcome Score − 12 short form; WOMAC: Western Ontario and McMaster Universities Osteoarthritis Index; SF-36: Short-Form 36; PCS: Physical Component Summary subscales of SF-36; MCS: Mental Component Summary subscales of SF-36

### Reproducibility

**Fig. 2:**
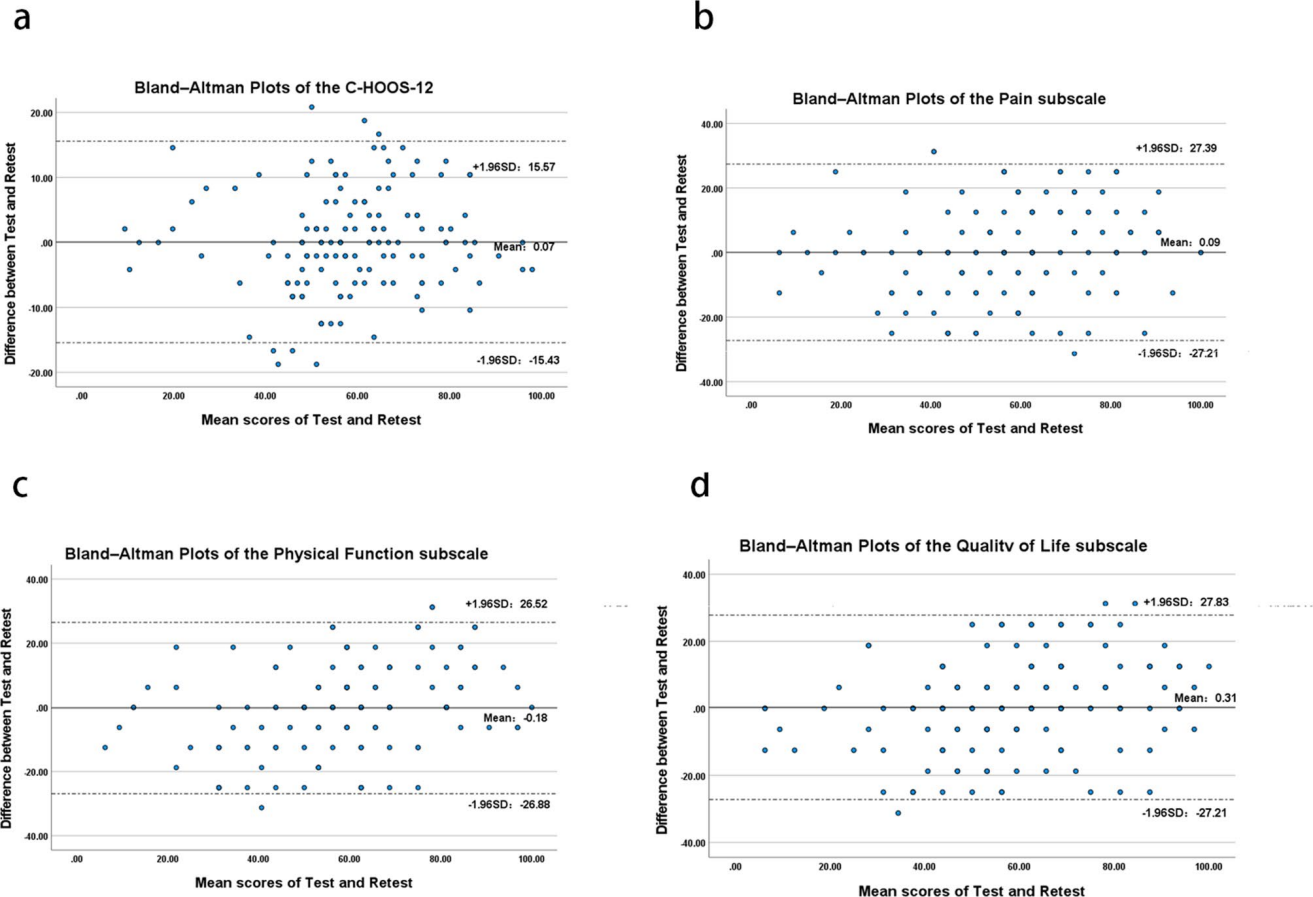
The Bland-Altman plot of test-retest reliability of the scale of the CHOOS scale and its sub-scales

In this study, the SEM value of CHOOS-12 was 5.46, indicating that the individual and group (in this study) score changes were the smallest and could be interpreted as true changes with MDC values of 15.13 and 1.27 respectively. The ICC value of the CHOOS-12 scale ranged from 0.859 to 0.925, suggesting that the scale has good test-retest reliability (Table 4). Additionally, the Bland-Altman plot also showed that there were no significant systematic errors in the CHOOS-12 questionnaire in the first two surveys (Fig. 2).

### Responsiveness

The scale scores of the CHOOS-12 and its subscales are shown in Table 8. The ES and SRM of CHOOS-12 are 2.08 and 2.42 respectively, indicating that the responsiveness of CHOOS-12 is good.

**Table 8:** Responsiveness related data of the C-HOOS-12

	C-HOOS-12	Subscales		
		Pain	Physical Function	Quality of Life
Mean ± SD (T0) ^a^	59.6 ± 17.3	59.1 ± 20.5	60.0 ± 18.0	59.8 ± 19.4
Mean ± SD (T2) ^a^	23.7 ± 14.5	19.9 ± 15.9	26.2 ± 16.5	25.1 ± 19.4
ES	-2.08	-1.91	-1.87	-1.79
SRM	-2.42	-2.16	-2.36	-2.19

^a^ The T0 was conducted at the beginning of this research and the T2 was conducted six months after the THA, the sample size included in the responsiveness analysis was 109 cases

C-HOOS-12: Chinese version of Hip disability and Osteoarthritis Outcome Score – 12 Short Form; ES: effect size; SRM: standardized response mean

## Discussion

THA is a surgical procedure aimed at correcting joint deformities, alleviating pain, restoring normal lower limb alignment and function, and improving the overall quality of life for patients. Therefore, THA can be performed on any patient experiencing hip joint deformity, pain, or functional impairment [37]. In addition to fractures, tumors, and infections, hip joint diseases include HOA, ONFH, and rheumatoid arthritis, among others. Studies indicate that the global prevalence of hip osteoarthritis is 0.85% [38], while the prevalence of rheumatoid arthritis in China ranges from 0.3% to 0.5% [39], The incidence of Perthes disease is between 1 in 400 and 1 in 35,000 [40], and the prevalence of ankylosing spondylitis in China ranges from 0.03% to 1.8% [41]. In our collected sample, 52 patients had osteoarthritis, and 85 had ONFH, accounting for 95.7% (137/143) of the total patient population. In contrast, cases of rheumatoid arthritis, Perthes disease, and ankylosing spondylitis were rare, with 2, 3, and 1 case(s), respectively (Table 1). Thus, the demographic composition of this study largely aligns with the epidemiological trends of hip joint diseases. During the validation of the original HOOS-12, the researchers only collected data from patients with hip OA. However, this does not mean that the use of HOOS-12 is limited to patients with HOA. At the beginning of the development of HOOS-12, the researchers’ aim was to obtain a tool that could comprehensively and specifically assess the condition of the hip joint (without specifying that it was caused by HOA) [17]. This is the basis for us to expand the applicable population to patients with chronic hip joint diseases, and other countries’ validation studies of HOOS-12 also included patients with other chronic hip joint diseases, such as Germany [20] and France [19].

Before the surgery, we calculated the ceiling effect of the CHOOS-12 scale and its sub-scales, and found that they were all less than 15%. In Australia, it was 1%, 3%, 2%, and 8% [21] ; in the UK, it was 0.5%, 1.4%, 1.1%, and 11.6% [18] ; in Germany, it was 5.3%, 0.2%, 0%, and 0% [20] ; in France, the HOOS-12 was 0% [19]. The floor effect of the total CHOOS-12 scale and its sub-scales was 0, while the floor effects in Australia, the UK, and France were all less than 1%. Only Germany had a slightly higher floor effect in the QOL subscale, at 8.5%. It can be seen that although the ceiling/floor effects measured in these countries have increased to some extent, they have not exceeded the maximum threshold of 15%, indicating that the HOOS-12 can still accurately distinguish the conditions of different patients when used in countries with different language backgrounds, and the content validity is good. However, we observed that at 6 months after the surgery, the ceiling effect of the CHOOS-12 scale was 0, which was significantly different from the research results of other countries (Australia: 17% [42]、English-speaking countries: 11.6% [43]、German-speaking countries: 11.9% [42] German-speaking countries: 11.9% [21]). A study on the use of analgesic drugs after THA in foreign countries in 2020 showed that 26.1% of patients were still using morphine-based analgesic drugs for pain relief one year after the surgery [44], while China has strict control measures and treatment time limits for analgesic drugs such as morphine and non-steroidal anti-inflammatory drugs (NSAIDs), and the use of opioid drugs for postoperative analgesia is generally 1–3 days after THA, while NSAIDs is 3–7 days, and the degree of pain may be closely related to the recovery of the patient’s functional status and quality of life after the surgery [45]. Therefore, the use of postoperative analgesic drugs may be the main reason for the significant difference in the ceiling effect of HOOS-12 in different countries.

The internal consistency of CHOOS-12 is the same as that of the aforementioned countries, and it also has excellent internal consistency. However, the Cronbach’s α value of the PF sub-scale in CHOOS-12 is 0.671. This might be related to the fact that this sub-scale only contains 4 items. Studies have shown that a smaller number of items will lead to a lower Cronbach’s alpha. Therefore, we conducted an analysis of the item-total correlation coefficient after correcting the items of this scale, and the corrected item-total correlation (CITC) of item 4 (whether it is difficult for you to walk on an uneven ground) of the PF subscale was 0.313, which was lower than 0.4. After deleting this item, the Cronbach’s α coefficient of the PF scale could be increased to 0.703 [46, 47] At the same time, confirmatory factor analysis showed that the factor loading of this item was also low (0.391). These evidences indicate that in CHOOS-12, item 4 is inconsistent with the overall concept of the PF subscale. Considering that the measurement of “whether it is difficult for you to walk on an uneven ground” by item 4 is a core and independent functional aspect in the daily life of hip joint patients, which has important clinical significance, this study decided to retain this item to ensure the completeness of the scale content. Further investigation is needed to examine the expression of this item or its applicability in a cross-cultural context.

The CFA results of this study showed that the pain scale had a good fit, while the PF and QOL sub-scales had poor fit. Therefore, we further reported the standardized factor loadings of the three sub-scales and their combined validity (CR). The results showed that except for the PF sub-scale, the CR of the other scales were all greater than 0.7. The validation results of the cross-cultural translated scale were different from the original version, which suggests that the measurement function of the scale in the new cultural context may have changed. This phenomenon is very common in scale validation, especially in the cross-cultural adaptation of classic theoretical scales [48]. There are many reasons for this. Firstly, the sample size is small (*N* < 200), which may increase the probability of error in the CFA results [31]. Although the sample size of this study has reached the standard for CFA, for a scale with only 4 items, the smaller the sample size, the less stable the CFA results. The sample size of the original HOOS-12 for validation exceeded 1000, and the results showed good construct validity. This may be one of the reasons for the poor structural validity of CHOOS-12. Therefore, although the total score and the pain subscale performed well, the dimensional structure of PF and QOL was unstable in the current sample and requires further investigation. Secondly, cultural differences can also lead to instability in the construct validity. During the validation process, we found that for item 4 of the PF subscale, both the internal consistency and the standardized factor loading were below the minimum value. This indicates that in the Chinese context, this item is unable to accurately reflect its relationship with functionality [48]. Chronic hip joint diseases that develop to the end stage will lead to loss of hip joint function and subsequently disability. A study on the depression status of disabled elderly people in China showed that 52.75% of the disabled elderly people had a stable trend in their epidemiological study depression scale (CES-D) score, always remaining in a non-depressed state (< 12 points). This to some extent suggests that in the Chinese population, most elderly people may not care about hip joint dysfunction after old age, so item 4 of the QOL scale cannot effectively represent its dimension. In the item options of the pain and QOL sub-scales, the fourth and fifth options have clear expressions of frequency or degree of “every day/anytime/never”, while in the PF sub-scale, the fourth and fifth options only have the general adjectives “very” and “extremely”. In the Chinese language environment, these two adjectives represent similar degrees and have no specific explanations or corresponding scenarios. Patients may confuse these two dimensions when choosing, resulting in a lower overall alpha coefficient of the functional status sub-scale, and thereby affecting the structural validity of the scale [26]. This suggests that Chinese researchers or clinicians, when using the CHOOS-12 scale to measure patients with chronic hip joint diseases, need to provide clearer explanations for the items in the PF sub-scale.

We used OHS, WOMAC and SF-36 as comparison scales to test the convergent validity and discriminant validity of CHOOS-12. However, different countries obtained different Spearman correlation coefficients due to the different comparison scales they used. The comparison scales used in France included OHS, HOOS, HOOS-JR and HOOS-PS. The items of these scales were all highly related to hip joint diseases, so the Spearman correlation coefficients of the French version of HOOS-12 were all > 0.8. Researchers in Germany and Australia both used the EuroQol5-Dimension5-LevelQuestionnaire (EQ-5D-5 L) scale [49], which showed a moderate correlation result with the HOOS-12 scale. The original HOOS-12, like CHOOS-12, also used the SF-36 universal scale as a comparison, and both showed a moderate correlation in the results.

Finally, we compared the scale results before and after THA treatment. The results showed that the CHOOS-12 had good responsiveness. However, this study has not yet determined the minimum clinically important difference (MCID) of this scale. The necessary prerequisite for determining the MCID is that the scale has good responsiveness. Then, through anchoring method or stepwise method, the MCID of CHOOS-12 in the Chinese population can be further determined, thereby converting statistical differences into clinically interpretable information. Currently, improving the clinical interpretability of patient-reported outcome indicators (PROM) has become an important trend. However, due to the different standards of different scales, their MCIDs are also different, which poses challenges for cross-study comparisons and syntheses. Therefore, Ramadanov proposed a standardized method based on MCID, converting the results of different PROMs in the hip arthroscopy pain field into a unified “MCID unit”, so as to achieve a meta-analysis that is both clinically meaningful and statistically comparable [50], providing very scientific methodological guidance for other PROM studies in the future. This study strictly verified the responsiveness of CHOOS-12 and laid a necessary foundation for determining the MCID of this tool.

## Conclusion

CHOOS-12 demonstrated good reliability and validity in this study. In terms of construct validity, the pain subscale performed well, but the PF and QOL models did not fit well. However, the application and interpretation of CHOOS-12 also need to consider several limitations of this study. Firstly, sample size is a key factor affecting the robustness of CFA results. Compared with the development and validation studies of the original scale, the sample size collected in this study is relatively limited, and a smaller sample size may pose a challenge to the credibility of the assessment of construct validity, such as causing unstable model fit indices and wider confidence intervals for parameter estimates (such as factor loadings), thereby affecting the validity of the statistical conclusion and the reproducibility of the model. This means that the results observed under the current sample need to be further confirmed for their stability in a larger sample. Secondly, the data in this study came from a single center. China is a vast country with diverse cultures, covering numerous ethnic minorities and regional cultures. Even with standardized Chinese translations, certain expressions may have subtle differences in understanding and response methods among people in different regions, cultures, or dialect backgrounds. This potential cultural difference may limit the generalizability of the research results to a wider Chinese population. Therefore, to establish the validity and reliability of CHOOS-12 in the Chinese population, future validation studies need to include larger-scale, multi-center samples from different geographical regions and cultural backgrounds. Finally, this study mainly focuses on the classical measurement attributes and has limited exploration of the MCID. In the future, the use of the “MCID unit” method framework can further convert these statistical changes into indicators with clear meanings for both patients and clinicians. Such an approach not only enhances the power of statistical tests but also enables a deeper examination of the measurement equivalence of the CHOOS-12 scale in different subgroups, ensuring that it becomes a truly reliable and universal tool for hip joint function assessment in the Chinese language usage environment.

## Data Availability

The data used in the analysis of this study are available from the corresponding author.
